# Neurological complications and infection mechanism of SARS-CoV-2

**DOI:** 10.1038/s41392-021-00818-7

**Published:** 2021-11-23

**Authors:** Dandan Wan, Tingfu Du, Weiqi Hong, Li Chen, Haiying Que, Shuaiyao Lu, Xiaozhong Peng

**Affiliations:** 1 Laboratory of Aging Research and Cancer Drug Target, State Key Laboratory of Biotherapy, National Clinical Research Center for Geriatricts, West China Hospital, Sichuan University, No. 17, Block 3, Southern Renmin Road, 610041 Chengdu, Sichuan PR China; 2 National Kunming High-level Biosafety Primate Research Center, Institute of Medical Biology, Chinese Academy of Medical Sciences and Peking Union Medical College, Yunnan, China; 3 State Key Laboratory of Medical Molecular Biology, Department of Molecular, Biology and Biochemistry, Institute of Basic Medical Sciences, Medical Primate Research Center, Neuroscience Center, Chinese Academy of Medical Sciences, School of Basic Medicine Peking Union Medical College, Beijing, China

**Keywords:** Diseases of the nervous system, Infectious diseases

## Abstract

Currently, SARS-CoV-2 has caused a global pandemic and threatened many lives. Although SARS-CoV-2 mainly causes respiratory diseases, growing data indicate that SARS-CoV-2 can also invade the central nervous system (CNS) and peripheral nervous system (PNS) causing multiple neurological diseases, such as encephalitis, encephalopathy, Guillain-Barré syndrome, meningitis, and skeletal muscular symptoms. Despite the increasing incidences of clinical neurological complications of SARS-CoV-2, the precise neuroinvasion mechanisms of SARS-CoV-2 have not been fully established. In this review, we primarily describe the clinical neurological complications associated with SARS-CoV-2 and discuss the potential mechanisms through which SARS-CoV-2 invades the brain based on the current evidence. Finally, we summarize the experimental models were used to study SARS-CoV-2 neuroinvasion. These data form the basis for studies on the significance of SARS-CoV-2 infection in the brain.

## Introduction

The emergence of Corona Virus Disease 2019 (COVID-19) in December 2019 was as a result of the outbreak of a novel human coronaviral pathogen, which was termed severe acute respiratory syndrome coronavirus 2 (SARS-CoV-2). This virus has a high similarity with severe acute respiratory syndrome coronavirus (SARS-CoV). The virus, SARS-CoV-2, belongs to a beta genus of coronaviruses and is the seventh member of human coronaviruses,^1,2^ sharing ~80% sequence similarity to SARS-CoV.^3,4^ Furthermore, the homology of SARS-CoV-2 is over 90% similar to those of coronaviruses from bats and pangolins, demonstrating its powerful ability to transmit cross-species.^5^ The virus shares a similar spherical structure to other coronaviruses (CoVs) with a diameter of ~100 nm and is a single-stranded positive-sense RNA virus,^6,7^ composed of four proteins: membrane (M) glycoprotein, nucleocapsid (N) protein, spike (S) glycoprotein, and envelope (E) glycoprotein.^8^ The N protein conjugates with genomic RNA to form nucleocapsid, while S, M, and E proteins combine to create an envelope to enclose the nucleocapsid.^6^ It was revealed that SARS-CoV-2 binds the angiotensin-converting enzyme 2 (ACE2) receptors under in the presence of the S protein and transmembrane protein serine protease 2 (TMPRSS2) could infect target cells expressing ACE2 receptors,^9–12^ including alveolar cells, macrophages, endothelial cells, kidney cells, intestinal epithelial cells, monocytes, neurons, glial cells, and neuroepithelial cells.^13–16^ Interestingly, some reports discovered that S protein of SARS-CoV-2 contains specific domains encoding polybasic cleavage sites^3^ and SARS-CoV-2 exhibits a high affinity for human ACE2 receptor,^14,17^ which may contribute to its stronger transmissibility and higher virulence compared with other CoVs.^18,19^

SARS-CoV-2 has spread fast throughout the world after the first COVID-19 case was detected in Wuhan, infecting a huge number of people. As of May 2021, according to the World Health Organization, there were over 163 million clinically confirmed cases with over 3.3 million COVID-19-associated deaths worldwide. COVID-19 was initially defined as a respiratory infection with fever, fatigue, abnormal chest X-ray, cough, and shortness of breath.^20–22^ In addition, a high proportion of COVID-19 patients exhibit neurological affectations during infection, such as hypogeusia, dizziness, headaches, myalgia, impaired consciousness, hyposmia, seizures, and ataxia.^23,24^ SARS-CoV-2 is extensively evidenced to cause many neurological diseases,^25–28^ similar to neurological manifestations previously reported for other respiratory viral infections,^29–31^ however, neurological symptoms of COVID-19 are highly frequent and disabling.^32,33^ In early investigations of COVID-19-positive patients in Wuhan, it was demonstrated that 36.4% displayed neurological manifestations, 8.9% presented PNS symptoms, the most prevalent of which was anosmia (5.1%).^34^ In particular, in severe COVID-19, partial neurodegeneration, brain edema, even encephalitis was observed.^35–37^ The neurodegenerative changes of cell death, hyperphosphorylation, and dislocation of Tau protein were documented in SARS-CoV-2-infected cells.^10^ Moreover, some COVID-19 patients were positive for SARS-CoV-2 in the cerebral spinal fluid (CSF) and brain tissue.^38–40^ These findings imply that SARS-CoV-2 infections are not restricted to the respiratory systems, but they also can reach the CNS and induce neurological conditions.^41^

However, the neuroinvasive mechanisms of SARS-CoV-2 remain unknown. COVID-19 is highly aggressive and is accompanied by hypoxia, abnormal clotting, and severe inflammation, so most CNS symptoms are identified as manifestations of peripheral pathologies. Concurrently, ACE2 and TMPRSS2 are vital for SARS-CoV-2 invasion and spread in the body, expressing suppressed levels in the brain,^42^ confirming that CNS symptoms are indications of peripheral pathologies. However, current research reveals that SARS-CoV-2 also can invade cells via Neuropilin-1 (NRP1),^43,44^ BASIGIN (BSG),^45^ Cathepsin L (CTSL), and furin^46^ which have a higher and broader expression in the brain when compared to TMPRSS2 or ACE2.^47^ This would be one mechanism through which SARS-CoV-2 propagates in the brain. Recent research has indicated that olfactory sensory neurons are a potential route for CNS invasion.^48–51^ It is clear that pathological inflammation, as well as the cytokine storm initiated by COVID-19 outside the brain, impacted the CNS.^52–55^ Understanding the invasion mechanisms of SARS-CoV-2 in the nervous system is critical for the rational treatment of patients. Although the SARS-CoV-2 neuroinvasion mechanism is yet unknown, and considering the highly similar viral sequence from SARS-CoV, a similar neuroinvasiveness mechanism may be relevant for SARS-CoV-2.^41^ Herein, we review previously documented SARS-CoV-2-associated CNS and PNS complications and further discuss the varieties of potential neuroinvasiveness mechanisms to help neurologists better understand SARS-CoV-2 influence on the nervous system and facilitate diagnosis as well as reasonable COVID-19 treatment.

## Neurological symptoms of COVID-19

**Fig. 1:**
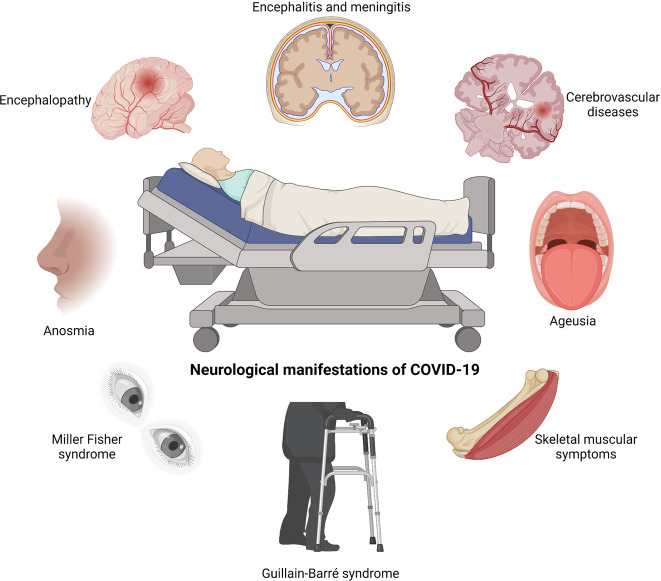
SARS-CoV-2-associated neurological symptoms. A variety of neurological manifestations are present in COVID-19 patients, such as encephalitis, encephalopathy, ageusia, anosmia, Miller Fisher syndrome, and Guillain-Barré syndrome

SARS-CoV-2 is extensively evidenced to present potential neuroinvasion. As a result, we reviewed studies on COVID-19, whose neurological manifestations could be assigned into three categories: CNS symptoms, PNS symptoms, and relative skeletal, muscular symptoms^34,56–58^ (Fig. 1 and Table 1).

**Table 1:** Neurological symptoms in COVID-19 patients

Neurological symptom	Affected region (reference)	Percentage (reference)
Acute cerebrovascular disease	Cerebral vessels^59,60^	2.8%^34^
Meningitis/encephalitis	CSF^39,84^	Case report^287^
Acute hemorrhagic necrotizing encephalopathy	Temporal lobe^288^	Case report^288,289^
Posterior reversible encephalopathy syndrome	Cortex^91,290,291^	Case report^91,290,291^
Demyelinating lesion	Spinal cord^292^	Case report^292^
Seizure	Left temporoparietal lobe^293–295^	0.5%^34^
Ischemic stroke	Cortex^34^	2.8%^34^
Dizziness	Whole brain^296^	9.4%^297^ 16.8%^34^
Headache	Whole brain^34,298,299^	3.4%^300^ 6.5%^297^ 13.1%^34^
Ataxia	Whole brain^34^	0.5%^34^
Impaired consciousness	Whole brain^34^	7.5%^34^
Brain edema	Brainstem^301^	Case report^301^
Anosmia	Olfactory neurons^126^	5.1%^34^
Ageusia	Tongue nerves^106,107,302,303^	5.6%^34^
Dysopia	Optic nerves^34^	1.4%^34^
Guillain-Barré **s**yndrome	Peripheral nerve demyelination^304–310^	Case report^310,311^
Miller Fisher syndrome	Whole brain^312,313^	Casa report^312,313^
Myalgia-muscle pain	Neuromuscular junction^314,315^	Case report^314,315^
Rhabdomyolysis	Muscle^316^	Case report^316^

## Central nervous system diseases and/or symptoms

Cerebrovascular disease is a group of diseases characterized by damage to brain tissues due to intracranial blood circulation disturbances, which generally occur in the brain’s blood vessels. As we know, virus infections can lead to vascular endothelial as well as vascular system damage, resulting in an overactive inflammation response, which can lead to ischemic and hemorrhagic infarcts and then further develop to thrombosis and vasculitis.^59,60^

Although the primary manifestation of COVID-19 is lung disease, cerebrovascular disease is also a primary neurological complication of COVID-19 that is associated with a high mortality rate.^61^ A retrospective study suggested that the severe COVID-19 patients generally had acute cerebrovascular diseases.^34^ The pooled prevalence of SARS-CoV-2-associated acute cerebrovascular disease is estimated to be 2.3%.^62^ An epidemiological and clinical characteristics study revealed that 52% of COVID-19 patients exhibited increased IL-6 levels, with 86% among them presenting high CRP levels, indicating a substantive inflammatory response.^63^ Concurrently, inflammation is required in developing cerebrovascular diseases, leading to cerebrovascular events.^64^ A Spanish medical center documented that among 1683 hospitalized COVID-19 patients, 1.4% developed cerebrovascular disease, in which 73.9% of them had ischemic stroke, and 21.7% had hemorrhagic stroke with increased levels of ferritin.^65^ Brain biopsies revealed thrombotic microangiopathy as well as endothelium injury, indicating that due to coagulopathy, seriously infected patients are highly vulnerable stroke.^66^ Meanwhile, additional researches have also corroborated this perspective. For instance, examining severe patients indicated that these people developed coagulation dysfunctions and high D-dimer levels,^67^ implying that those people may be at an increased risk of venous thrombosis accompanied by hemorrhagic stroke.^68^ A 75-year-old COVID-19-related woman who had no notable pre-disposing factors but with severe bipartite pneumonia as well as acute pulmonary embolism implies that serious infections are an induced factor of acute venous embolism and associated stroke.^69,70^ During COVID-19 dissemination, patients with cardiocerebrovascular risk factors are more susceptible to develop acute stroke.^71^ Six COVID-19 patients had an acute ischemic stroke in large vessels with typical signs of neurological dysfunctions, including aphasia, prosopoplegia, sensory loss, dysarthria, and acute confusion. Two patients exhibited multiple infarctions attributed to coinstantaneous venous as well as arterial thrombosis. Five cases were positive for lupus anticoagulants, demonstrating a coagulation disturbance. Angiograms showed occlusion of cerebral artery trunk or branches.^72^ In the other six cases, four patients were diagnosed with ischemic stroke, while the remaining two patients presented with hemorrhagic stroke. They all experienced severe pneumonia and complications in multiple organs, and high transaminase and lactate dehydrogenase (LDH) levels, indicating poor clinical outcomes. However, before COVID-19 outbreak, only one patient had a possible vascular risk factor associated with stroke.^73^ Four SARS-CoV-2-infected patients from New York demonstrated that two of them developed subarachnoid hemorrhage (SAH) as well as hemorrhagic transformation following ischemic stroke, but SARS-CoV-2 remained undetectable in the CSF.^74^ SARS-CoV-2 easily attacks the lung, lowering blood-oxygen saturation and then causing hypoxemia, resulting in altered consciousness, delirium or confusion, and intracerebral hemorrhage or acute/subacute stroke.^63,70,75^ Moreover, SARS-CoV-2 impact on ACE2 receptor imbalances the renin–angiotensin system (RAS), resulting in microcirculation disorders, impairing the regulation of cerebral blood flow, and producing an excessive rise in blood pressure, hence increasing the risk of encephalorrhagia and ischemic stroke.^76,77^ Hemorrhagic lesions may arise from coagulation dysfunctions caused by severe systemic infections or by invasions of the vascular endothelium by viruses.^78^ As a result of cerebral white matter damage and blood vessels, the general state of CIVD-19 patients may aggravate.^66^ The stress state caused by injuries to endothelial cells and hypoxia can promote inflammation, hypercoagulability, and pulmonary encephalopathy.^79^

Although it is seemly hard for the virus to penetrate the CNS, CoVs can invade the CNS own their neurotropism;^80,81^ then may lead to overreacting immune responses and induce fatal encephalitis and meningitis. Encephalitis refers to pathogen-induced inflammatory lesions (neuronal damage and nerve tissue injury) in brain parenchyma, with typical symptoms, including elevated temperature, headache, vomiting, fatigue, and consciousness disorders.^82^ Based on autopsy analyses of 17 cases, eight patients exhibiting cerebral edema and vascular congestion were found SARS-CoV-2 positive in brain tissue.^40^ Another autopsy report also indicated edema in brain tissue and neuronal degenerations in SARS-CoV-2-infected people.^83^ Meanwhile, a Japanese male with developed seizure as well as unconsciousness after fever and general weakness exhibited a SARS-CoV-2 positivity in the CSF and was diagnosed with SARS-CoV-2-related meningitis.^39^ McAbee et al. presented an encephalitis case with positive SARS-CoV-2 in CSF, accompanied by increased red and white cells.^38^ The team of Beijing Ditan Hospital evidenced SARS-CoV-2 positivity in the CSF of SARS-CoV-2-infected patients by genome sequencing, hence verifying viral encephalitis.^84^ As known, immune cells and viruses can enter the brain via a weakened blood–brain barrier (BBB).^85^ Even though not all patients test positive for SARS-CoV-2 in CSF, symptoms of nervous system infections and injuries implies the possibility of COVID-19-related neurological complications.^57^

Acute necrotizing encephalopathy is a rare severe explosive encephalopathy and that is common as a result of viral infection. The pathogenesis of which is generally considered as cytokine storm-induced breakdown of blood–brain barrier.^54^ Encephalopathy is recognized as the most common CNS complication of COVID-19, caused by hypoxia or systemic diseases.^86^ Statistically, ~50% of hospitalized patients with COVID-19 have encephalopathy,^87^ with common signs including dizziness, cognitive dysfunction, ataxia, mental disorder, or even impaired consciousness. Old people with hepatic, cardiovascular, and renal comorbidities, as well as immunosuppressed persons, are more likely to develop encephalopathy following COVID-19 infection.^34^ A 50-year older woman was the first to be diagnosed with acute necrotizing hemorrhagic encephalopathy associated with COVID-19, with primary symptoms of fever, altered mental state, and cough, and neuroimaging indicated symmetrical multifocal changes as well as thalamic invasions.^88^ Reichard et al. reported that a COVID-19 patient appeared to die of acute disseminated encephalomyelitis according to pathological findings.^89^ Meanwhile, some people manifest unexpected seizures owing to high fever. Combined with analysis of patients previously found to have epilepsy, apprehension and sleep disorders caused by the COVID-19 pandemic may be an additional trigger for epilepsy.^90^ Four SARS-CoV-2-infected patients aged 60–70 years presented with seizures and exhibited elevated blood pressure and renal injury, systemic inflammation, and hypercoagulopathy and were diagnosed with posterior reversible encephalopathy syndrome (PRES).^91^ Moreover, autopsy analyses of four confirmed COVID-19 cases revealed hypoxia-induced cerebral impairments and observed small perivascular lesions that appeared in the white matter.^92^ However, there is no clear evidence to indicate whether neuropathologic lesions are caused by primary vascular diseases that are a result of damage to white matter or demyelinating diseases through a by-infectious mechanism.

## Peripheral nervous system symptoms

The SARS-CoV-2 virus can cause chemosensory disorders, including anosmia, ageusia, hyposmia, etc.^5,93,94^ Over one-third of confirmed COVID-19 patients exhibit smell or taste disturbances,^62^ which can develop prior to, contemporaneously, or after fever and cough.^95^ Approximately 50% of patients experience anosmia or ageusia in the early stages of COVID-19.^96,97^ It is also reported that about 11.8% of COVID-19 cases manifested an olfactory disorder before developing other symptoms, implying that anosmia is crucial for early disease detection.^98^ Statistics indicate that anosmia and ageusia can be regarded as COVID-19 predictors. When combined with fever, discrimination accuracy can increase up to 75%.^99^ Although the prevalence of dysosmia and dysgeusia decreases in older people,^100^ female patients have higher smell disturbance.^101^ Another research indicated that about 12% of SARS-CoV-2-infected patients develop only anosmia as their initial symptom.^57^ A clinical study analysis with 33 COVID-19 cases reported that ~63.6% of patients developed chemosensitive dysfunction, and 13 patients presented combined anosmia and ageusia.^102^ Moreover, patients may exhibit other related sensory dysfunctions, such as tinnitus, sore throat, vertigo, dysphagia, and hearing.^103,104^ These studies indicate that SARS-CoV-2 virus can cause hyposmia and anosmia, implying that thorough examination of otolaryngologic manifestations may assist in early COVID-19 diagnosis.

Guillain-Barré syndrome (GBS), a PNS condition, is induced by overactive immune system, which attacks the PNS by mistake.^105^ The initial signs are tingling and weakness in the periphery, but they can rapidly distribute leading to whole-body paralysis. Representative neurological manifestations include progressive dysergia, flaccid paralysis, and areflexia. The first case of COVID-19-related GBS was a 61-year-old woman from Wuhan, China.^106^ The initial symptom was the weakness of the lower limbs which is in line with the neurological examination: areflexia and symmetric weakness in both legs and feet. Various examination results revealed lymphocytopenia, thrombocytopenia, elevated protein levels in CSF, and motor and sensory nerve demyelination, all of which were suggestive with GBS. Another GBS patient was a 71-year-old male in Italy who had no history of neurological disorder.^107^ Although brain CT scan revealed normal findings, electroneurography measurements indicated the absence of tibial nerve compound muscle action potential (CMAP) and sural nerve sensory nerve action potential (SAP), indicating peripheral neuropathy caused by demyelination, a typical feature of GBS. Toscano et al. documented five COVID-19-related cases with limb or facial paresis as well as paresthesia within some days after the appearance of COVID-19 symptoms.^108^ MRI results revealed caudal roots or facial nerve enhancement, indicating that nerves undergo immune response. To date, a rapid increase of GBS cases has been confirmed in COVID-19 patients.^109,110^ However, a previous epidemiological cohort study found no relationship between GBS and COVID-19.^111^ It is unclear whether the causative agent for GBS is SARS-COV-2 virus itself or other infections secondary to COVID-19-related patients, therefore, more studies are required to elucidate on this phenomenon.

SARS-CoV-2 infection can result in dyspnea and decrease the blood-oxygen saturation by attacking lung.^63^ Indeed, severe COVID-19 patients with acute respiratory distress syndrome (ARDS) can cause serious systemic hypoxemia, which may be related to congestion and edema observed in brain tissue.^34,112^ Meanwhile, hypoxemia induced by ARDS and lung injury may promote SARS-CoV-2 to invade the brain tissue. A study about three COVID-19 patients without breathing difficulties under low oxygen tension has been reported.^113^ These patients had extremely low blood-oxygen levels, which would result in unconscious or multiple organ failures. However, all of them were awake and had no signs of dyspnea, challenging the understanding of basic biology nowadays. Notably, patients with hypoxemia may exhibit mild symptoms but may rapidly progress to multiple organ failure and death.^114^ Another research indicated that severe systemic hypoxemia would aggravate dementia symptoms in AD patients infected with COVID-19.^115,116^ To date, various hypotheses have been advanced to account for hypoxemia. One is that SARS-CoV-2 may directly act on oxidation-sensitive receptors, alter respiratory center responses to low oxygen levels, and modify the way PCO2 to blunt the response of the brain to hypoxia.^113^ Another hypothesis indicates that in a corticolimbic network, SARS-CoV-2 can cause neuronal damage that may alter the secretion of endogenous neuropeptides or neurotransmitters, which are related to perceptual effects.^117^ More studies are needed to confirm or disprove these views in the future.

## Skeletal muscular symptoms

Skeletal muscular symptoms are another prevalent PNS complication of COVID-19 patients, presenting tiredness, myositis, myalgia, and skeletal muscle injury. Several cases of skeletal muscular symptoms in SARS-CoV-2-positive people have been reported.^34^ Research of 213 COVID-19 cases indicated that ~85.2% of patients had significantly elevated serum creatine kinase. Another published study demonstrated elevated lactate dehydrogenase and creatine kinase levels, which may be caused by skeletal muscle injury.^118^ Mehan et al. described nine COVID-19 cases who had backache, dyskinesia, and paresthesia in lower limbs. Seven patients exhibited intramuscular edema through spinal cord MRI, confirming paraspinal myositis.^119^ In addition, most coronavirus infections could result in functional defects as well as myalgias in skeletal muscles with increased CK levels.^120^ Similar skeletal muscular symptoms were found in the studies of SARS-CoV.^121^ Virus-induced elevated pro-inflammatory cytokines level might be another reason to aggravate the muscular injury.^122^ However, additional research should be conducted to validate whether SARS-CoV-2 can cause skeletal muscular sequelae in the long term.

## Mechanisms through which SARS-CoV-2 invades the nervous system

Generally, the CNS with a very intricate brain barrier system to defend against the virus invasion, including blood-cerebrospinal fluid barrier, blood–brain barrier (BBB), and brain-cerebrospinal fluid barrier. Although the CNS is protected by multilayer barriers, where also can be invaded by various viruses involved in the glial cell or neuronal invasions.^123^ Coronaviruses were reported to reach the CNS causing neurovirulence.^124^ However, the exact mechanism of coronaviruses invade the CNS has not been fully distinct.^125^ Mostly, viral infections start from peripheral tissues and then spread to the peripheral nerves and finally reaches the central nervous system.^126^ This process may explain the presence of neurological lesions, like demyelination.^127^ ACE2 is highly expressed on vascular endothelial cells, and also expressed on olfactory epitheliums, striatum, cortex, substantia nigra, as well as the brainstem,^128^ suggesting SARS-CoV-2 can directly infect vascular endothelial cells to cross the BBB and then can infect cells throughout the CNS. The ACE2 receptor is also expressed on glial cells and neurons of various structures, including olfactory epitheliums, striatum, cortex, substantia nigra, as well as the brainstem, implying that SARS-CoV-2 has the potential to infect cells in the CNS. Moreover, in COVID-19 patients, hyposmia is a frequent complication, indicating the infection of the olfactory nerve. Therefore, the synaptic connections via olfactory nerves would be another mechanism through which SARS-CoV-2 enters the CNS. SARS-CoV-2-induced inflammation is also considered to disrupt the BBB allowing virus to enter the CNS.^129,130^

## CNS expression of key viral infection factors

### Expression of ACE2 and TMPRSS2

Currently, it is accepted that SARS-CoV-2 gains entry into cells via ACE2^9,24^ under the participation of TMPRSS2^49,131^ due to the high expression of both these two proteins in the lung. In addition, numerous studies indicate that both ACE2 and TMPRSS2 also expressed in the brain at suppressed levels (Table 2), so in theory the virus should infect brain cells.^14,49,132–134^ Lazartigue and coworkers, according to early immunohistochemical work, found the existence of ACE2 in the neurons of rat brain rather than glia.^133^ They also established that ACE2 is critical in blood pressure regulation and in autonomic nerve system diseases. ACE2 is expressed both in tractus solitarius and in the areas involved in blood pressure central regulation, such as paraventricular nucleus.^135^ Interestingly, this ACE2 is expressed in a professional group of CNS structures without the BBB, named circumventricular organs, implying a possible direct path for brain invasion by SARS-CoV-2. Recent RNA-Seq studies have found that ACE2 is elevated in the substantia nigra, ventricles, olfactory bulb, posterior cingulate cortex, posterior cingulate cortex, choroid plexus, middle temporal gyrus, frontal, and motor areas (https://www.proteinatlas.org/). ACE2 was also found to be expressed in postmortem frontal cortex vessels with different calibres and significantly elevated in the brain vasculature of dementia and hypertension patients.^136^ SARS-CoV-2 was found to invade neurons and then cause their necrosis in the study of human ACE2 transgenic mice as well as brain organoids;^137,138^ however, the amounts of affected cells were limited due to low TMPRSS2 and ACE2 levels. The autopsy studies on 32 COVID-19 patients indicated thrombotic and thromboembolic signs in olfactory mucosa and CNS observed from the section of olfactory mucosa and thalamus samples, and SARS-CoV-2 S protein also observed in the endothelial cells of small CNS vessels. The levels of virion load in olfactory mucosa were 124% higher than in the lower respiratory tract,^139^ demonstrating that nasal epithelium may be an entry point for SARS-CoV-2 to reach the brain through the centripetal route. Notably, no ACE2 mRNA was observed in the human brain.^140,141^ To date, ACE2 distribution mostly relies on mRNA data analysis, but mRNA incompletely reflects the distribution of true functional protein. Therefore, numerous immunohistochemical characterization researches are urgently required.

**Table 2:** Receptors or proteins related to SARS-CoV-2 infection in the nervous system

Receptor or protein	Main expression region	Reference
ACE2	Pituitary gland, nucleus accumbens, hypothalamus	^42,317–321^
TMPRSS2	Pituitary gland, hypothalamus, cerebellum	^49,322,323^
NRP1	Olfactory bulb	^43^
BASIGIN	Frontal cortex, pituitary gland	^45,145–147^
Cathepsin L	Pituitary gland, spinal cord	^45,143,144^
Furin	Lung, brain	^46^
ATR1	Pituitary gland, substantia nigra	^324–326^

### Other receptors expressed in the brain

Based on the above description, ACE2 expression in the brain is very low. However, numerous COVID-19 patients had higher levels of viral modifications in brains than predicted by ACE2 expression patterns alone, implying the presence of alternative viral entry mechanisms besides ACE2. More recently, neuropilin-1 (NRP1) was identified as a novel mechanism of access to the brain for SARS-CoV-2, which was expressed in brains and olfactory bulbs (OBs) (Table 2), at high levels relative to ACE2 and TMPRSS2.^43,44,142^ The autopsies of the olfactory epithelium from COVID-19 patients determined that the infected olfactory epithelial cells with high expression of NRP1.^43^ In addition to NRP1, SARS-CoV-2 invades cells via BASIGIN (GBS) and Cathepsin L (CTSL), which also facilitated SARS-CoV-1 to infect cells^45,46,143–147^ (Table 2). All these proteins have a higher expression in the human brain when compared to ACE2 or TMPRSS2 and express in the olfactory bulb. Accordingly, SARS-CoV-2 can reach the brain possibly through olfactory epithelium (OE), vagus nerve, BBB, or CSF and infect the brain.

### Transcribial route and neuronal transport dissemination

**Fig. 2:**
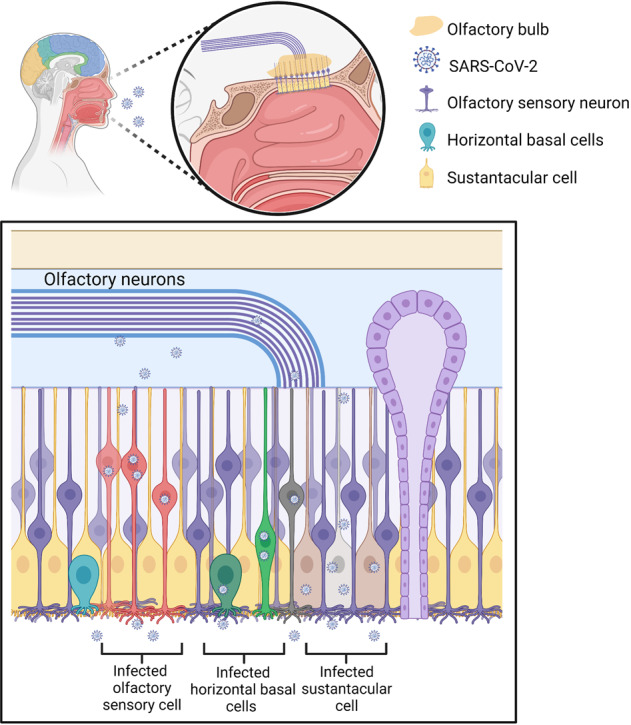
SARS-CoV-2 may invade the brain through the olfactory nerve. SARS-CoV-2 infects the olfactory epithelium via the ACE2 receptor. The olfactory epithelium surrounds horizontal basal cells with ACE2 receptor. Human horizontal basal cells express ACE2, suggesting they can be infected by SARS-CoV-2. Horizontal basal cells can further mature into olfactory neurons. We propose that infected horizontal basal cells can mature into SARS-CoV-2-infected olfactory neurons. These infected olfactory neurons share a synaptic connection with neurons in the olfactory bulb (OB). This may allow for viral spread from the periphery into the CNS. The OB has many connections throughout the brain. This allows for rapid viral transit to many areas of the brain

Numerous evidence indicated that certain CoVs initially invaded peripheral nerve terminals and then spread throughout CNS through anterograde/retrograde of synapses,^41,83,148^ including HEV67,^148,149^ as well as OC43-CoV.^29^ In the PNS, the olfactory nerve is the main route for SARS-CoV-2 to invade CNS due to high levels of TMPRSS2 and ACE2 in olfactory epithelium cells, both of which are required for viral binding and accumulation.^125,131,150,151^ The olfactory nerve is a CNS conduction bundle instead of a real nerve, which directly contacts the brain (Fig. 2).^152,153^ In the nasal cavity, the olfactory mucosa is composed of neurons, Bowman’s glands, basal cells, and epithelial cilia.^154–156^ The special olfactory neuroepithelium of the nasal cavity has an apical surface composed primarily of sustentacular cells.^157^ Support cells were demonstrated to present high levels of TMPRSS2 and ACE2,^49^ indicating that they were predisposed to SARS-CoV-2 infection.^48^ As observed in the hamster, OE infection would spread to horizontal basal cells (HBCs) and then to immature or mature olfactory neurons.^51^ Meanwhile, the infected HBCs subsequently matured into OSNs, which would reach OB via a synaptic path that may potentially infect CNS.^158^ Through this way, a peripheral infection could access OB and further spread throughout the brain. Another autopsy study presented that SARS-CoV-2 invaded CNS by traversing the neural mucosal and subsequently penetrated the neuroanatomical areas with olfactory tract projections, such as respiratory as well as cardiovascular control centers in the medulla.^139^ The authors also observed the characteristic CoV substructures and SARS-CoV-2 RNA in olfactory epithelium cells and olfactory mucus cells. The viral load in olfactory mucosa, medulla oblongata, olfactory tubercle, oral mucosa, olfactory bulb, trigeminal ganglion, and cerebellum were assessed by means of RT-qPCR.^139^ The human ACE2 transgenic mice were intranasally administered with SARS-CoV-1, and the related antibodies evaluated in OB, parts of cortical areas, as well as the basal ganglia ~60–66 h later.^159^ Four days after first exposure, the infection was spread through most OB and distributed to most areas of the brain as well, including the hypothalamus, pons, medulla, thalamus, midbrain, amygdala, basal ganglia, hippocampus, and cortex.^159^ These data indicate that OB is a route for coronavirus CNS infection. In addition, in the model of hamster, SARS-CoV-2 was observed to infect OB and the brain.^48,50,51,160^ Moreover, our previous research demonstrated that SARS-CoV-2 primarily invades the CNS via OB in rhesus monkey and subsequently viruses quickly spread to other areas of the CNS, including the hippocampus, medulla oblongata, and thalamus.^161^ Although the exact mechanism of early CNS invasion remained unclear, when these results are combined, it is plausible that SARS-CoV-2 infection can spread to the brain after it reaches OB. In this condition, it is speculated that SARS-CoV-2 infection spreads from the olfactory epithelium to the olfactory bulb and then to the olfactory nerve, applying endocytosis and exocytosis for trans-synaptic transfers^28,162^ (Fig. 2).

In addition to the olfactory nerve, SARS-CoV-2 may employ other potential peripheral nerves to reach the brain, including the trigeminal, vagus, and nasopharyngeal nerves. Anatomically, the vagus nerve is a part of the enteric nervous system and connects to gastrointestinal tracts with elevated expressions of NRP1 and ACE2. Both ACE2 and TMPRRSS2 are expressed in intestinal enteric neurons and glia, indicating that they are susceptible to SARS-CoV-2.^163^ The gut–brain axis is a critical component to cause CNS disorders.^164^ A study of 42 COVID-19 patients found that ~66.67% of them were positive for SARS-CoV-2 RNA in feces.^165^ In vitro, SARS-CoV-2 was shown to infect human intestinal epithelium.^166^ The virus would spread from duodenal cells to the brainstem neurons via anterograde and retrograde transmission.^167^ Consequently, it is reasonable that SARS-CoV-2-infected enterocyte would spread to neuronal and glial cells of enteric nervous systems and finally invade CNS through the vagus nerve.^125,168^ In this route, various data showed that initial SARS-CoV-2 infection in the lung would cause subsequent viral spreading to the brain, specifically the areas of the thalamus and brainstem, including the vagus nerve medullary nuclei.^169^ Matsuda et al. documented that, through the vagus nerve, influenza A virus spread from the respiratory tract to the vagal ganglia.^170^ Another study demonstrated ACE2 expression of the vagal complex in rodents.^171^ However, the data in humans are scarce, and more studies are required to disseminate SARS-CoV-2 vagus nerve. Similarly, trigeminal and nasopharyngeal nerves may be another route for SARS-CoV-2 to reach the brain because both are exposed to the virus. Aoyagi et al. described a COVID-19 patient who developed dysphagia and observed dysregulated pharyngolaryngeal sensations, mesopharyngeal contractile dysfunction, and silent aspiration through the video endoscopy, high-resolution manometry, and videofluorography implying possible infections of the trigeminal or nasopharyngeal nerves.^172^ In addition, studies should evaluate the expressions of ACE2, TMPRRSS2, NRP1, and other related proteins to confirm whether these mechanisms can cause CNS infections in COVID-19 patients.

### Hematogenous route

The hematogenous route is a possible route for SARS-CoV-2 to enter the brain as it includes virus circulation into the bloodstream.^123,173,174^ In this condition, BBB is a common entry route for virus spread to CNS. Through the hematogenous route, SARS-CoV-2 invades the CNS through two mechanisms: the infection of vascular endothelial cells to cross BBB and the induction of inflammatory responses to disrupt BBB.

## SARS-CoV-2 infects vascular endothelial cells and crosses the BBB

**Fig. 3:**
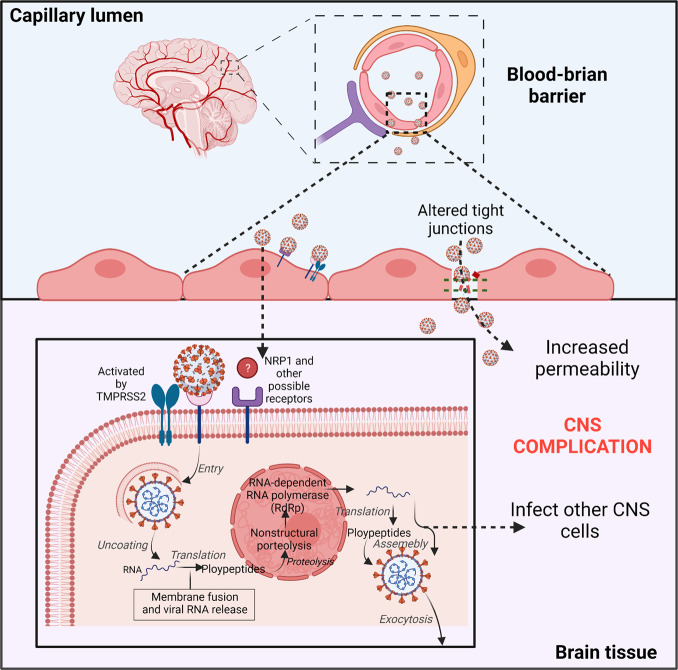
SARS-CoV-2 possibly directly infects vascular endothelial cells via the ACE2 or NRP1 receptors. Viral particles in the bloodstream can reach the brain through the blood–brain barrier (BBB) by infecting and replicating inside brain microvascular endothelial cells. Infection of neurons by SARS-CoV-2 and the increased BBB permeability could be responsible for severe neurological symptoms in COVID-19

According to reports, infection and injury of epithelial barrier cells enable the virus to access the lymphatic system and bloodstream and further spread to various organs, such as the brain.^28^ Autopsy analyses of lungs of five COVID-19-confirmed patients revealed that viral proteins were observed in lung capillaries and after SARS-CoV-2 infection led to endothelial necrosis and capillary damage.^175^ SARS-CoV-2 causes damage to lung blood vessels.^175,176^ Although only a small number of COVID-19 patients have been shown to be positive for SARS-CoV-2 in blood, it suggests that the virus reaches the bloodstream and possibly infects other organs, like the brain.^177^ Once the virus has gained access to the bloodstream, it can rapidly infect the endothelial cells in the vasculature due to expressions of ACE2, TMPRSS2, and NRP1^42,178^ (Fig. 3). Moreover, a structural analysis of the postmortem examination of a COVID-19 patient indicated that viral particles presented in neural as well as capillary endothelial cells of the frontal lobe tissue, implying that virus can access the brain by infecting vascular endothelial cells.^16^ Autopsy analyses of three COVID-19 patients revealed that SARS-CoV-2 could infect endothelial cells, and endotheliitis were detected in both lungs, kidney, heart, small intestines, and liver.^179^ Additionally, an in vitro study in human blood vessel organoids demonstrated SARS-CoV-2 invasion and replication, corroborating the mechanism by which infected brain endothelial cells allow blood-borne viruses to enter the brain.^180^ Generally speaking, due to the presence of BBB, infections do not frequently occur in the brain, even when viruses or bacteria are present in the bloodstream. BBB comprises neurons, pericytes, astrocytes, vascular smooth muscle cells, and endothelial cells.^181^ The brain microvascular endothelial cells (BMVECs) are a major BBB component. The main function of BBB is to protect the brain by preventing hematogenous entry of pathogens and neurotoxic compounds into the brain.^182^ Consequently, the virus needs to pass blood–brain barrier and infect the brain through the hematogenous route. Previous research confirmed that ACE2^42^ and NRP1^178^ are expressed in human BMVECs, indicating that they may be potential SARS-CoV-2 targets (Fig. 3). To date, the evidence about SARS-CoV-2 infecting BMVECs of the BBB are limited; only one autopsy study described the viral-associated proteins detected in BMVECs of the frontal lobe of a COVID-19 patient.^16^ This shows that SARS-CoV-2 infects BMVECs of the BBB, even though this infection from the brain or blood is unclear. Meanwhile, in vitro, JHM OMP1, a kind of coronavirus, was shown to infect isolated BMVECs from humans and rhesus macaques,^183^ indicating that some coronaviruses can invade BMVECs. Choroid plexus exhibited a highly permeable blood–CSF barrier than BBB and was found to express ACE2 and TMPRSS2,^163^ implying that it may be another potential route through which the virus invades the CNS. According to a study conducted on a human choroid plexus model, SARS-CoV-2 not only infected the choroid plexus cells but also destroyed the blood–CSF barrier, providing another route for the virus to enter the brain.^184^ All these data show that coronavirus can directly invade as well as replicate in vascular endothelial cells and cross the BBB (Fig. 3).

## SARS-CoV-2 initiated systemic inflammatory responses to disrupt the BBB

SARS-CoV-2 may also gain entry into the CNS via inflammation, which disrupts the blood–brain barrier. Even though it has not been conclusively demonstrated that SARS-CoV-2 infection results in inflammation that allows viral entry into CNS, we offer three probable mechanisms based on existing research.

## SARS-CoV-2 initiates cytokine secretion by immune cells

**Fig. 4:**
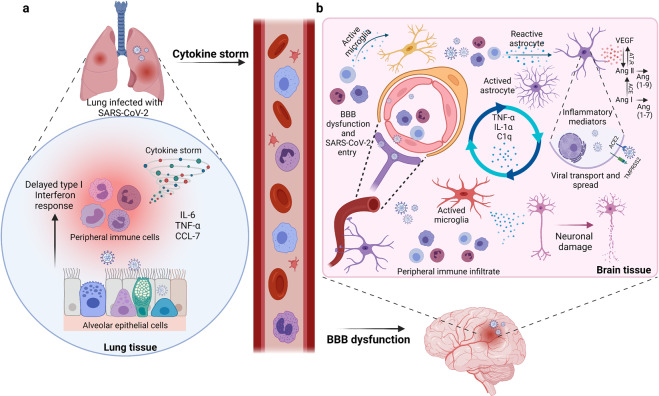
SARS-CoV-2 infection can cause excessive peripheral immune responses to result in BBB dysfunction. **a** The cytokine storms with remarkable BBB permeability effects may allow the virus or infected immune cells reach the brain. **b** Possible CNS pathological mechanisms caused by the severe peripheral hyperinflammation associated with COVID-19. The infected immune cells invade in brain and release cytokines to activate glial cells to release pro-inflammatory cytokines and VEGF which could cause severe neurological symptoms in COVID-19

Immune responses resulted from a viral infection can cause nervous system damage. It is worth mentioning that SARS-CoV-2 has the ability to infect immune cells, which may subsequently invade CNS. SARS-CoV-2 was demonstrated to activate various immune cells, including macrophages/monocytes, T cells, neutrophils, and natural killer cells (Fig. 4). These activated immune cells, in turn, could kill the virus through cytokine release,^185–189^ including interleukin (IL), interferon (IFN), tumor necrosis factor (TNF), and chemokine.^129^ In normal physiological conditions, pro-inflammatory factors and immune cells can form a positive feedback cycle to keep the balance of cytokines.^185^ However, SARS-CoV-2 infection can cause excessive immune responses, triggering a systemic inflammatory response due to cytokine storms, and then mainly cause damage to blood vessels^112,173^ (Fig. 4). The cytokine storms with remarkable BBB permeability effects may allow the virus or infected immune cells to reach the brain, promoting related CNS symptoms.^129,130^ It has been demonstrated that infected peripheral lymphocytes and macrophages act as dissemination vehicles to facilitate the pass across BBB, meninges, and choroid plexus.^173,190^ SARS-CoV-2 was reported to primarily infect human monocytes, whereas MERS-CoV was found to infect both T cells and monocytes. Meanwhile, SARS-CoV-2 was determined to infect dendritic cells. However, both monocytes and macrophages presented low ACE2 expression, implying that an unknown mechanism might exist in communications between the host innate immune response and SARA-CoV-2. The exact mechanism by which SARS-CoV-2 infects immune cells remains unknown.

## Activated glial cells secrete pro-inflammatory cytokines

Several neurotropic viruses were demonstrated to infect glial cells, causing them to become pro-inflammatory and secrete various cytokines.^191^ As mentioned above, ACE2 is expressed on glial cells (Fig. 4). Concurrently, glial cells are a component of BBB. Therefore, it may be that SARS-CoV-2 entered in CNS via hematogenous route, olfactory nerve, or other mechanisms, which also possibly infect glial cells and cause a pro-inflammatory state^84,192^ (Fig. 4). Moreover, Lechien et al. observed numerous inflammatory factors produced by glial cells after SARS-CoV-2 infection, including interleukin-6, interleukin-15, tumor necrosis factor α, and so on.^98^ In turn, these cytokines can disrupt BBB, enabling the virus to enter the brain and finally induce symptoms of CNS diseases. To date, there are few reports on glial cells of COVID-19 patients. Whether SARS-CoV-2 invades glial cells via binding to ACE2 or by other glial cell receptors remains unclear. Additional research is required to demonstrate SARS-CoV-2 effects on glial cells.

## Vascular endothelial growth factor initiates inflammatory responses

Vascular endothelial growth factor (VEGF) with a wide distribution in CNS mainly regulates angiogenesis, endothelial cell proliferation, and vascular permeability.^193^ It has been reported that astrocytes express VEGF and FIt-1 following CNS trauma and that inflammatory cells are the main source of VEGF in the injured CNS.^194–196^ In addition, the renin–angiotensin system was activated following SARS-CoV-2 binding to ACE2, which was relevant to the inflammation response, and then through the combination of angiotensin II (Ang II) and angiotensin II type 1 receptor (AT1R) to promote VEGF synthesis. In most brain diseases, VEGF was found to cause inflammatory responses to disrupt BBB rather than promoting angiogenesis alone.^193,197^ Angiogenesis is invariably associated with inflammation, resulting in increased vascular permeability and inflammatory cell recruitment^197^ (Fig. 4). ACE2 can respectively catalyze Ang I and Ang II to Ang 1–9 and Ang 1–7.^198^ Since SARS-CoV-2 binds to ACE2, an active enzyme can boost the signaling of the ACE/Ang II/AT1R axis, resulting in excessive Ang II production. Excessive Ang II promoted the growth of ACE2 in the SARS-CoV-2-infected brain. Consequently, VEGF further enhanced Ang II, indicating a vicious circle in releasing pro-inflammatory cytokines, including IL-8, IL-6, TNF-a, and IL-1b^193,199^ (Fig. 4). Among these cytokines, IL-6 is an essential member of the pro-inflammatory cytokine family, inducing various proteins correlated with acute inflammation.^200^ In addition, the hyper-inflammatory syndrome of COVID-19 was reported to mainly involve IL-6.^201^ IL-6 was deemed as an indication of respiratory failure in hospitalized COVID-19 patients.^202^ Besides, another research indicated that severe COVID-19 symptoms are positively correlated with IL-6 levels. As a result, IL-6 may be considered as a predictive marker of COVID-19 severity.^84,188^

## Experimental models for nerve system studies

Experimental models should be thoughtfully selected due to the complex interactions between the host and SARS-CoV-2. The rational use of animal models enables us to obtain reliable scientific data and address problems of interest. Although many animals and humans may exhibit similar physiological, pathological, or even therapeutic responses to the same diseases, it is essential to keep in mind that variations among species can lead to incorrect conclusions.^203^ Therefore, it is critical to establish a connection between human disease and model.^204^ To understand the exact mechanism through which SARS-CoV-2 invades the nerve system, animal or cellular models that can mimic the symptoms as well as pathological processes of SARS-CoV-2 nerve infection patients is urgently needed. Here, we summarize the current in vivo and in vitro experimental models applied to study SARS-CoV-2 infection of the nervous system.

## CNS cell lines

Most research methods of SARS-CoV-2 currently available are based on SARS-CoV due to their 78% nucleotide homology.^205^ Although the neurotropism of SARS-CoV-2 has been demonstrated,^206^ no specialized neural cell line models have been applied to study it. Human-induced pluripotent stem cells (hIPSCs), such as neurons, microglia, and neural progenitor cells (NPCs), have been employed as in vitro models to evaluate nervous infections of SARS-CoV-2.^138,207^ The HOG cell line is obtained from human oligodendroglioma and is usually used to study neurons, while the cell line C6 derived from glioma is used to study neural susceptibilities to SARS-CoV-2 infection.^208–211^ However, both of these two cell lines exhibit low viral replication levels compared with other susceptible cell lines, such as Caco-2 or Vero E6.^212^ Meanwhile, human H4 brain neuroglioma cells, CHME-5 human fetal microglia cell line, LA-N-5 human neuroblastoma cell line, as well as U-87 MG and U-373 MG astrocytic lines have been employed to study CNS virulence abilities of HCoV-OC43 and HCoV-229E.^213–218^ In addition, oligodendrocytes, microglia, human primary neurons, and astrocytes have been utilized to investigate these various viruses.^219,220^ Therefore, nerve cell lines are possibly appropriate to study SARS-CoV-2, which requires additional research in the future.

## Brain organoids as SARS-CoV-2 CNS infection model

As in vitro 3D culture systems derived from self-organizing stem cells, organoids are composed of multiple cell types and can imitate the physiological conditions of corresponding human organs such as architecture, functioning, and genetic signature.^221,222^ In addition, organoids can overcome the limitations of cell culture systems, including the incapacity to study cell–cell interactions. As a result, organoids are suitable models to study various human physiological or pathological processes, including infection, neurotropisms, and possible treatments for SARS-CoV-2.^222–224^ Although infections by SARS-CoV-2 mainly cause lung injury, other organs like the liver, kidney, cardiovascular system, and nervous system are also affected.^225–227^ Currently, human lung, liver, kidney, intestine, and blood vessels organoids have been deployed to study SARS-CoV-2 infection.^138,180,228–230^ Human brain organoids have been applied to study SARS-CoV-2 CNS infections. In these brain organoids, viruses were observed to mainly infect mature cortical neurons, and neurodegenerative effects were also detected in SARS-CoV-2-infected cells, such as hyperphosphorylation, cell necrosis, and Tau protein mislocation.^207^ Furthermore, these authors discovered no procreative virus replication in the first 4 days after infection, corroborating the postulate that CNS may be a long-term SARS-CoV-2 reservoir.^231^ Bullen et al. detected an increased viral titer in neural cells between 6 and 72 h post-brain organoid infections with SARS-CoV-2, implying an active viral replication in neural cells during initial days.^232^ Not only were these viral particles found in the neuronal soma, but also in the neurites.^232^ Mesci et al. also observed SARS-CoV-2 infection in neurons, including mature cortical neurons and NPCs, as well as cell death associated with the injury of excitatory synapses.^233^ Moreover, this study evaluated the efficiency of Sofosbuvir in treating SARS-CoV-2 infection, which is a brain-penetrant antiviral drug for RNA virus approved by FDA.^234^ The results demonstrated that viral accumulation and neuronal death were decreased after Sofosbuvir treatment in brain organoids.^233^ Similarly, Song et al. described the neuroinvasive capacity of SARS-CoV-2 in brain organoids, especially in NPCs and mature cortical neurons.^235^ Hypermetabolic state in the infected cells and the accumulation of viral particles in endoplasmic reticulum-like structures were both observed, implying virus replication in neural cells. Furthermore, severe SARS-CoV-2-infected areas accompanied by a hypoxic environment and massive neuronal death indicated that SARS-CoV-2 infection causes the death of nearby neural cells.^235^ Finally, IgG antibodies against SARS-CoV-2 from CSF of patients with COVID-19 were demonstrated to block SARS-CoV-2 invasion in human brain organoids.^235^ In summary, SARS-CoV-2 is neuroinvasive and may further cause CNS diseases. Concurrently, these studies also reveal that human brain organoids are optimal in vitro model for studying SARS-CoV-2 infection in CNS.

## Animal models

Due to the interconnected and intricate connections between various organs of the human body, the exact mechanism of SARS-CoV-2 invasion can only be understood through systemic interaction between virus and host. Although various cell lines and organoids as in vitro models are faster systems to study CNS infection of SARS-CoV-2, these studies are only limited to specific cell types and organs. Consequently, the in vivo models are critical for exploring the complex pathophysiology of SARS-CoV-2. To date, several animal models have been used to investigate SARS-CoV-2 brain infection, including mouse, hamster, ferret, and non-human primates. SARS-CoV-2 infectivity varies due to differences in the capacity of the virus to combine with various ACE2 species.^236^ Therefore, in these in vivo models, it is critical to adopt appropriate approaches to identify SARS-CoV-2 presence in tissues and explore the advantages and limitations of these methods. Plaque formation indicates virus replication but not cellular localization. PCR reveals RNA information rather than cellular localization and virus replication, and whether subgenomic RNA implies the active replication remains unknown.^237^ Antibodies against viral spike protein or nucleocapsid demonstrate cellular localization but cannot differentiate the whole virus from cleaved proteins in the brain.^238^ In situ hybridization provides both tissue localization and viral RNA.^238^

SARS-CoV-2 exhibits low infectivity to wild-type mice; therefore, to study the infectivity and spread of SARS-CoV-2 in this species, either mice must be engineered to express human ACE2, or SARS-CoV-2 virus must be mice-adapted.^239,240^ Currently, multiple engineered mouse models have been established to study SARS-CoV-2 invasion.^240–244^ SARS-CoV-2 can infect the olfactory epithelium of mice expressing human ACE2 (hACE2), and whether infection further progresses to the brain is likely dependent on promoter type, which controls hACE2 expression. Previous researches demonstrated that mild infection symptoms and few SARS-CoV-2 viral particles were detected in brains of mice models expressing hACE2 when promoters were exogenous or endogenous murine ACE2 or cytomegalovirus (CMV).^137,244–246^ In these models, the evidence for the presence of SARS-CoV-2 mostly based on PCR,^247,248^ but did not prove through in situ hybridization or immunocytochemistry. The K18-hACE2 mice were generated to study SARS-CoV-1 several years ago, their expressions of hACE2 were controlled by K18 cytokeratin. It has been reported that these mice models are frequently fatal, possibly due to infections of the brain,^137,243,244,249–254^ but the new mice models constructed through CRISPR/Cas9 and knock-in methods do not exhibit neuroinvasion.^239^ Human ACE2 expression replaces endogenous ACE2 expression in these new mouse models, which are physiologically realistic than K18-hACE2 mice, but the utilized adenoviral vector may occasionally provoke host responses.^242^ Numerous viruses, including mouse hepatitis virus, human coronavirus OC43 (HCoV-OC43), and herpes simplex virus, have previously been reported to readily infect olfactory neurons and further spread to secondary and tertiary olfactory brain centers.^29,158,255–258^ However, in the new mice models and wild-type animals, few SARS-CoV-2 infections were detected in olfactory circuits, may be due to a deficiency of virus entry proteins in olfactory neurons. Viruses with strong neuroagression exhibit a common characteristic of high expression of their entry proteins in infected neurons.^29,158,256–258^ The studies in mice models using human ACE2 expression that monitored the arrival time of virus in different brain structures revealed that SARS-CoV-2 infection in olfactory bulbs was not more than other brain structures, such as the brainstem, hypothalamus, and thalamic nuclei.^249,253,254^ The limitation of engineered mice models is that hACE2 expression may not occur in the same cell types as expressed in humans, and may not have the same expression levels as well, and hence may not cause SARS-CoV-2 neuroinvasion in these mice even with normal ACE2 expression. Thus, experimental data from these mice must be analyzed prudently.^259^

Hamsters are considered better physiological animal models for exploring SARS-CoV-2 neuroinvasion since their ACE2 expression can cause medium-to-high SARS-CoV-2 infectivity, which is similar to that of humans.^51,236,259,260^ Seven researches using hamster models determined whether the virus could be present in the brain following nasal inoculation with SARS-CoV-2.^48,50,51,160,260–262^ Three of them revealed virus or viral RNA in the brain or olfactory bulb;^260–262^ however, four studies observed no brain infection.^48,50,51,160^ Using different viral titers might cause these differences between studies during infection. Indeed, different studies have demonstrated that viral titer varies up to 10000-fold, from 10 to 1 × 10^5^ plaque-forming units,^51,262^, and the virus was commonly detected in the brain when higher virus titers were employed during infection.^51^

Ferrets have been utilized as animal models to study the infection mechanism of various viruses, such as SARS-CoV or influenza.^263–265^ In addition, it has been proven that ferrets are sensitive to SARS-CoV-2 infection.^236,266,267^ In most studies the inoculation of SARS-CoV-2 was the intranasal route in ferrets.^268–270^ Viruses were observed in the brain in some ferrets, and viral antigen was detected in the nasal cavity, nasal respiratory, and olfactory epithelium.^267^ In ferret models, viruses were observed in the brain only through plaque formation or quantitative PCR, rather than immunocytochemistry, which caused difficulty in determining the cellular source of viruses. Therefore, more evidence are required to demonstrate SRAS-CoV-2 neuroinvasiveness in ferret models.

Since non-human primates have been employed to investigate Middle East respiratory syndrome coronavirus and SARS-CoV,^271–273^ these models were considered suitable for studying SARS-CoV-2 as well. Six studies surveyed SARS-CoV-2 infection in the brain following nasal or upper respiratory route inoculation. Three of them did not observe SARS-CoV-2 in the brain through quantitative PCR.^274–276^ Another three studies detected viral RNA in several brain regions.^161,277,278^ Recently, using rhesus monkeys, we found that SARS-CoV-2 can also invade the CNS via the olfactory route.^161^ In this study, SARS-CoV-2 viral protein was observed not only in the olfactory bulb but also in the thalamus, hippocampus entorhinal area, and medulla oblongata. Further research revealed that SARS-CoV-2 can invade the CNS but without efficient infection as well as replication.^161^ The viruses appeared rapidly in CSF, implying that neuronal transfers along olfactory nerves may not be the only pathway for SARS-CoV-2 to reach the brain and that alternative routes may exist.

Most viruses in humans were found in the olfactory epithelium, particularly in sustentacular cells.^43,139,260^ In some patients, the infections occurred in the brain, including the brainstem, thalamus, and hypothalamus.^139^ In some cases, viruses were also detected in the cerebral cortex, choroid plexus, and CSF,^139,163,238,279–283^ but not in CSF in other cases.^284,285^ However, all these SARS-CoV-2 neuroinvasion data in humans are obtained on the final outcome, but no time-course data. Overall, it is critical to select an appropriate animal model to better understand SARS-CoV-2 nerve invasion mechanism.

## Conclusion and perspectives

Although SARS-CoV-2 mainly causes pulmonary disorders, growing evidence demonstrate the possible neuroinvasion by SARS-CoV-2, as evidenced by isolating SARS-CoV-2 from CSF, SARS-CoV-2 discovery in the olfactory system, and various neurologic symptoms in patients with COVID-19. COVID-19 patients have been reported to exhibit CNS and PNS complications, including cerebrovascular diseases, encephalitis and meningitis, encephalopathy, anosmia and ageusia, Guillain-Barré syndrome, hypoxemia, and skeletal muscular symptoms. However, it is challenging to identify whether CNS symptoms resulted from CNS infection rather than peripheral origin due to hypoxia occurrence, blood clots, and cytokine storms in advanced patients. In addition, the SARS-CoV-2 neuroinvasion mechanism remains unclear. To date, the possible SARS-CoV-2 neuroinvasion mechanisms include invasion by the olfactory nerve, direct infection of vascular endothelial cells, and invasion through inducing inflammatory responses that disrupt the BBB. Indeed, other potential peripheral nerves, such as the trigeminal, vagus, and nasopharyngeal nerves, may also be potential routes for SARS-CoV-2 to reach the brain. All these pathways are associated with ACE2 or NRP1, therefore, the most effective approach to determine how SARS-CoV-2 invades CNS may be to identify the distributions of NRP1 and ACE2, as well as other possible receptors such as BSG in the human brain and the expression of these proteins in which brain cell types. This article summarized the possible SARS-CoV-2 neuroinvasive mechanisms based on the research of SARS-CoV and autopsy studies of SARS-CoV-2 patients. However, SARS-COV-2 is more complex and transmissible than SARS, so studies on SARS are not entirely applicable to SARS-COV-2. At the same time, the almost insurmountable obstacle of autopsy reports is the relatively long postmortem interval, especially in a pandemic situation of COVID-19. Analysis of these autopsy samples is affected by the well-known autolysis of cells and tissues. Therefore, we must also consider other SARS-CoV-2 invasion mechanisms until conclusive pathological evidence is discovered. Although various in vitro and in vivo models were employed to study SARS-CoV-2 neuroinvasion, most animal models do not accurately predict SARS-CoV-2 infection in CNS due to different distributions and density of ACE2, NRP1, BSG, and TMPRSS2 between these models and the human brain. Protein data indicate that low TMPRSS2 and ACE2 levels are expressed in human CNS, while rodents and humanized mice exhibit higher levels than humans.^159,286^ In addition, the value of using traditional immunohistochemistry analysis of brains from people who died due to COVID-19 may be limited. It may be effective to quantify the amounts of cells which express entry proteins. In short, more experimental studies are needed to unravel the precise neuroinvasion mechanisms of SARS-CoV-2, which may lead to find more efficient treatment strategies for these neurological complications.
